# Abnormal posterior semicircular canal function may predict poor prognosis in patients with severe and profound ISSNHL

**DOI:** 10.3389/fneur.2023.1123165

**Published:** 2023-01-30

**Authors:** Yang Yang, Dekun Gao, Xiaobao Ma, Jiali Shen, Qin Zhang, Xiangping Chen, Qing Zhang, Yulian Jin, Jianyong Chen, Maoli Duan, Jun Yang

**Affiliations:** ^1^Department of Otorhinolaryngology-Head and Neck Surgery, Xinhua Hospital, Shanghai Jiaotong University School of Medicine, Shanghai, China; ^2^Shanghai Jiaotong University School of Medicine Ear Institute, Shanghai, China; ^3^Shanghai Key Laboratory of Translational Medicine on Ear and Nose Diseases, Shanghai, China; ^4^Division of Ear, Nose and Throat Diseases, Department of Clinical Science, Intervention and Technology, Karolinska Institutet, Stockholm, Sweden

**Keywords:** severe and profound, idiopathic sudden sensorineural hearing loss, prognosis, vHIT, posterior semicircular canal

## Abstract

**Background:**

Severe and profound idiopathic sudden sensorineural hearing loss (ISSNHL) generally leads to unfavorable prognosis, and has a considerable impact on patient quality of life. However, related prognostic factors remain controversial.

**Objective:**

To elaborate the relationship between vestibular function impairment and the prognosis of patients with severe and profound ISSNHL, and investigated the relevant factors affecting prognosis.

**Methods:**

Forty-nine patients with severe and profound ISSNHL were divided into good outcome group [GO group, pure tone average (PTA) improvement > 30 dB] and poor outcome group (PO group, PTA improvement ≤ 30 dB) according to hearing outcomes. The clinical characteristics and the proportion of abnormal vestibular function tests in these two groups were analyzed by univariate analysis, and multivariable logistic regression analysis was performed for parameters with significant differences.

**Results:**

Forty-six patients had abnormal vestibular function test results (46/49, 93.88%). The number of vestibular organ injuries was 1.82 ± 1.29 in all patients, with higher mean numbers in PO group (2.22 ± 1.37) than in GO group (1.32 ± 0.99). Univariate analysis revealed no statistical differences between the GO and PO groups in terms of gender, age, side of the affected ear, vestibular symptoms, delayed treatment, instantaneous gain value of horizontal semicircular canal, regression gain value of vertical semicircular canal, abnormal rates of oVEMP, cVEMP, caloric test and vHIT in anterior and horizontal semicircular canal, however, significant differences were found in the initial hearing loss and abnormal vHIT of posterior semicircular canal (PSC). Multivariable analysis revealed that only PSC injury was an independent risk factor for predicting the prognosis of patients with severe and profound ISSNHL. Patients with abnormal PSC function had worse initial hearing impairment and prognosis than patients with normal PSC function. The sensitivity of abnormal PSC function in predicting poor prognosis in patients with severe and profound ISSNHL was 66.67%, specificity was 95.45%, and positive and negative likelihood ratios were 14.65 and 0.35, respectively.

**Conclusion:**

Abnormal PSC function is an independent risk factor for poor prognosis in patients with severe and profound ISSNHL. Ischemia in the branches of the internal auditory artery supplying the cochlea and PSC may be the underlying mechanism.

## 1. Introduction

Idiopathic sudden sensorineural hearing loss (ISSNHL) is defined as an otologic emergency in which three or more consecutive frequency hearing thresholds rise suddenly by 30 dB or more within 72 h, accompanied by tinnitus and concurrent or delayed vestibular symptoms in some patients (1). Previous studies have reported spontaneous recovery in a proportion of patients with ISSNHL (2); however, the outcome is often poor in some patients with severe-to-profound ISSNHL (3, 4). Severe and profound unilateral hearing loss may lead to speech communication impairment, particularly in noisy environments, and difficulty in localizing sound sources, which has a considerable impact on the long-term quality of life and mental status of patients (5, 6).

There is no consensus on the prognostic factors of ISSNHL and various factors have been identified including the degree of initial hearing loss, age at onset, presence of vestibular symptoms, classification of hearing loss, and time of intervention (7–9). Recent studies have found that the results of a series of vestibular function tests can predict the outcomes of patients with ISSNHL to some extent (10, 11). However, there are relatively few studies on the prognosis of patients with severe or profound ISSNHL. The purpose of this study was to elucidate the relationship between vestibular function impairment and the prognosis of patients with severe and profound ISSNHL, and to further investigate the relevant factors affecting prognosis.

## 2. Methods

### 2.1. Patients and study design

A retrospective study was performed on patients with ISSNHL who were hospitalized at the Department of Otolaryngology-Head and Neck Surgery, Xinhua Hospital, Shanghai Jiao Tong University School of Medicine between September 2020 and September 2022. This study was approved by the Institutional Ethics Committee of Xinhua Hospital, Shanghai Jiao Tong University (NM: XHEC-D-2022-259), and all patients provided consent for their data to be used for this research.

The following inclusion criteria applied: patients with unilateral ISSNHL, PTA (0.5, 1,2, 4 k Hz) ≥ 65 dB in the affected ear, and normal hearing in the opposite ear; intact tympanic and Type A tympanogram in both ears; complete patient medical record data and records of pure tone audiometry (performed on the first day of admission and the day before discharge), ocular vestibular-evoked myogenic potential test (oVEMP), cervical vestibular-evoked myogenic potential test (cVEMP), caloric test, and video head impulse test (vHIT). Exclusion criteria were as follows: a history of genetic disorders associated with familial deafness; sensorineural hearing loss secondary to noise exposure or ototoxic drugs; space-occupying lesions of the internal auditory canal, central organic pathology, and external and middle ear disease; a malignant tumor; inability to complete the course of treatment or audiology-vestibular function test due to liver or kidney disease or other reasons.

A total of 49 patients were included in this study. All enrolled patients underwent an audiology-vestibular function test on the first day of admission, and hearing was retested the day before discharge after one course of treatment. The treatment protocol was as follows: (i) daily intravenous dexamethasone 10 mg, (ii) daily intratympanic injection of dexamethasone (5 mg), and (iii) daily hyperbaric oxygen therapy. The completion of 10 days of treatment was considered completion of the course of treatment.

### 2.2. Audiometry

Pure tone audiometry was performed using the MADSEN Astera clinical diagnostic audiometry system (GN Otometrics, Denmark). The binaural PTA was taken as the mean of the four frequencies of 500, 1,000, 2,000, and 4,000 Hz. Severe hearing loss was defined as a pretreatment hearing level between 65 and 80 dB HL, and profound hearing loss was defined as a pretreatment hearing level ≥ 80 dB HL, according to the latest standards of the World Health Organization (12).

### 2.3. Vestibular function tests

#### 2.3.1. vHIT

An EyeSeeCam head tosser (Interacoustics Company) was used for the testing. The patient wore an eye patch containing a head velocity monitoring sensor and was placed in the sitting position with the head held still and the eyes focused on a fixed point (target point) 1.5 m in front of them. The examiner stood behind the patient and calibrated the target point before examining the horizontal and vertical semicircular canals in the conjugate plane of each of the three pairs of semicircular canals following the standard vHIT technique. The EyeSeeCamTM software objectively recorded the 60 ms instantaneous gain value of the horizontal semicircular canal, regression gain value of the vertical semicircular canal, asymmetry ratio of the three pairs of conjugate semicircular canals, and refixation saccades from the beginning to the end of the head impulse. Any one of the following conditions was considered abnormal: (i) an instantaneous gain value of the horizontal semicircular canal <0.8 and a regression gain value of the vertical semicircular canal <0.7; and (ii) 10 or more refixation saccades with a peak angular velocity >100°/s in 20 head impulses (13).

#### 2.3.2. Caloric test

The patient was placed in the supine position, with the head in forward flexion at 30° to ensure that the horizontal semicircular canal was perpendicular to the floor. Cold air (24°C) and hot air (50°C) were instilled into the patient's ears separately for 60 s each time. The patient's electronystagmogram was recorded for 1 min after instillation, with the interval between instillations being 5 min after the disappearance of the previous nystagmus. The average slow-phase velocity (SPV) during the strongest period of temperature-induced nystagmus was recorded, and canal paresis (CP) was calculated using the Jongkees formula, which reflects the asymmetry of the horizontal semicircular canal bilaterally. A CP value >25% was considered abnormal and indicated a relative reduction in ipsilateral horizontal semicircular canal function. The dominant preponderance ratio (DP) was calculated to determine the lateral preponderance of nystagmus, and a DP value >30% was considered abnormal.

#### 2.3.3. Air conducted sound cVEMP

The Biologic Navigator Pro Auditory Evoked Potential (Biologic Auditory Evoked Potential Software Ver.7.3.1, Denmark) was used to perform the test. The reference electrode was placed between the clavicular joints, the ground electrode was placed between the two eyebrows of the forehead, and the left and right test electrodes were placed in the middle of the sternocleidomastoid muscle on the left and right sides, respectively, with an electrode impedance of ≤ 5 kΩ. The stimulation signal was 500 Hz, with 90 dB nHL short tone bursts, 1 ms rise/fall time, 2 ms duration at peak, 5 Hz stimulation rate, and 50 superimposed times. The stimulation sound was delivered using air conduction insert earphones to elicit a VEMP response. The patient was instructed to lift the head off the pillow after hearing the unilateral stimulation sound and to elevate the head 30° in the supine position to keep the sternocleidomastoid muscle tense until the stimulation sound stopped, before returning to the original lying position.

#### 2.3.4. Air conducted sound oVEMP

The equipment and relevant parameters used for the testing were the same as those described above. The reference electrode was placed on the lower jaw, ground electrode was placed between the two eyebrows on the forehead, and test electrode was placed 1 cm below the central part of the contralateral eyelid. The patient was instructed to gaze upward after hearing the unilateral stimulation sound, keeping the eye position at 25–30° and blinking as little as possible to maintain the lower oblique muscle tone until the stimulation sound stopped.

The interwave amplitude of P1-N1 was recorded as the vertical distance between the apex of the N1 and P1 waves. The amplitude asymmetry ratio (AR) was calculated as the ratio of the absolute value of the difference between the amplitudes of the two sides to the sum of the wave amplitudes of the two sides. Abnormal VEMP was defined as a waveform not elicited or an AR of >29% (14).

### 2.4. Grouping according to therapeutic outcomes

According to the Chinese Medical Association of Otolaryngology criteria, the return of the hearing threshold of the damaged frequencies to normal, healthy ear, or pre-disease levels was considered complete recovery; partial recovery was defined as hearing improvement > 30 dB HL; slight recovery was defined as hearing improvement between 15 and 30 dB HL, and no recovery was defined as hearing improvement of <15 dB HL (15).

Based on the degree of hearing recovery, forty-nine patients with severe and profound ISSNHL were divided into two groups: the good outcome group (GO group, including complete and partial recovery, PTA improvement >30 dB) and the poor outcome group (PO group, including slight and no recovery, PTA improvement ≤ 30 dB).

### 2.5. Statistical analysis

SPSS software (version 26.0) was used to analyze the data. Measurement data were expressed as mean ± standard deviation (M ± SD), and count data were expressed as percentages. The Shapiro-Wilks test was performed to test the normal distribution of the measurement data, the independent *t*-test was used for data that was normally distributed, and the rank sum test was used for data that was not normally distributed. The differences between groups were analyzed using univariable logistic regression analysis, and those parameters with significant differences were analyzed using multivariable logistic regression analysis. Differences were considered statistically significant at *P* < 0.05.

## 3. Results

### 3.1. Clinical data for all patients

This study enrolled 49 patients (25 males and 24 females, 48.69 ± 18.63 years old) were enrolled in this study, with 17 right ears and 32 left ears. Twenty-six patients had vestibular symptoms at the time of the consultation (26/49, 53.06%). The mean timeframe between the onset of symptoms and treatment was 5.94 ± 4.64 (1–21 days). Severe hearing loss was observed in 14 ears and profound hearing loss was observed in 35 ears. The mean hearing threshold of the affected ear was 92.09 ± 18.05 dB HL before treatment and 66.76 ± 33.32 dB HL after treatment. Of the 46 patients who underwent abnormal vestibular function tests (46/49, 93.88%), 29 had abnormal oVEMP (29/49, 59.18%), 28 had abnormal cVEMP (28/49, 57.14%), 25 had abnormal caloric tests (25/49, 51.02%), and 23 had abnormal vHIT tests (23/49, 46.94%), including 19 (19/49, 38.78%) with abnormal posterior semicircular canal (PSC) function, 12 (12/49, 24.49%) with abnormal horizontal semicircular canal function, and one (1/49, 2.04%) with abnormal anterior semicircular canal function (Table 1).

**Table 1:** Clinical data of all patients included in this study.

**Variable**	**Statistical data (*N* = 49)**
**Gender**	
Male	25 (51.02%)
Female	24 (48.98%)
**Affected side**	
Left	32 (65.31%)
Right	17 (34.69%)
Age	48.69 ± 18.63
vestibular symptoms	26 (53.06%)
Onset of treatment (days)	5.94 ± 4.64
**Hearing loss**	
Severe hearing loss	14 (28.57%)
Profound hearing loss	35 (71.43%)
Initial hearing threshold (dB HL)	92.09 ± 18.05
Hearing threshold after-treatment (dB HL)	66.76 ± 33.32
**Hearing recovery**	
GO group	22 (44.90%)
PO group	27 (55.10%)
**Abnormal vestibular function tests**	46 (93.88%)
Abnormal oVEMP	29 (59.18%)
Abnormal cVEMP	28 (57.14%)
Abnormal caloric test	25 (51.02%)
Abnormal vHIT	23 (46.94%)
Abnormal horizontal canal	12 (24.49%)
Abnormal anterior canal	1 (2.04%)
Abnormal posterior canal	19 (38.78%)

### 3.2. GO group vs. PO group

#### 3.2.1. Clinical characteristics

There were 22 patients (12 males and 10 females, 45.91 ± 20.31 years old) in the GO group, including 16 left ears and six right ears, of which nine had vestibular symptoms at the time of the consultation. The mean hearing threshold of the affected ear was 85.00 ± 17.47 dB HL before treatment, and 38.18 ± 19.17 dB HL after treatment.

There were 27 patients (13 males and 14 females participants, 50.96 ± 17.20 years old) in PO group, including 16 left ears and 11 right ears, in which 17 patients had vestibular symptoms at the time of the consultation. The mean hearing threshold of the affected ear was 97.87 ± 16.67 dB HL before treatment, and 90.05 ± 22.32 dB HL after treatment. A comparison of clinical data between the two groups is shown in Table 2.

**Table 2:** Univariate and multivariate analysis of prognostic factors in patients with severe and profound ISSNHL.

	**Group**		**Univariate analysis**	**Multivariate analysis**	
	**GO group (*n* = 22)**	**PO group (*n* = 27)**	***P*-value**	***P*-value**	**Exp (B)**
Gender, male	12 (54.55%)	13 (48.15%)	0.656		
Age	45.91 ± 20.31	50.96 ± 17.20	0.344		
Affected side, left	16 (72.73%)	16 (59.26%)	0.327		
vestibular symptoms	9 (40.91%)	17 (62.96%)	0.127		
Onset of treatment (days)	6.09 ± 5.43	5.81 ± 4.00	0.835		
Initial hearing threshold (dB)	85.00 ± 17.47	97.87 ± 16.67	0.016	0.382	1.02
Abnormal cVEMP	10 (45.45%)	18 (66.67%)	0.139		
Abnormal oVEMP	14 (63.64%)	15 (55.56%)	0.568		
Abnormal Caloric test	10 (45.45%)	15 (55.56%)	0.483		
**vHIT**					
Abnormal horizontal canal	4 (18.18%)	8 (29.63%)	0.358		
Instantaneous gain value	1.11 ± 0.17	1.02 ± 0.19	0.111		
Abnormal posterior canal	1 (4.54%)	18 (66.67%)	0.001	0.002	33.009
Regression gain value	1.17 ± 0.21	0.98 ± 0.40	0.065		
Abnormal anterior canal	0 (0)	1 (3.70%)	1		
Regression gain value	1.29 ± 0.27	1.40 ± 0.32	0.191		

Exp (B), odds ratio.

#### 3.2.2. Vestibular function test

In the vestibular function test results in the GO group, the abnormality rate of oVEMP was the highest (14/22, 63.64%), followed by cVEMP (10/22, 45.45%), caloric test (10/22, 45.45%), horizontal semicircular canal (4/22, 18.18%), and PSC (1/22, 4.54%). No anterior semicircular canal dysfunction was observed.

**Figure 1:**
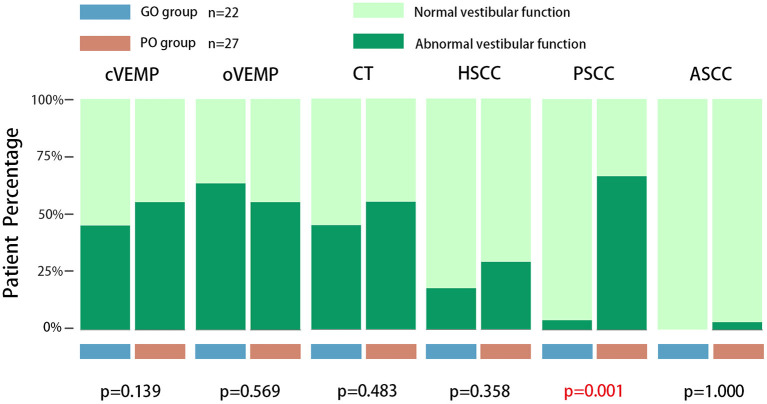
The comparison of vestibular function between the GO group and PO group. There was no significant difference in terms of the abnormal rates of oVEMP, cVEMP, caloric test, and vHIT in anterior and horizontal semicircular canals by univariate logistic regression analysis, and only a significant difference in the posterior semicircular canals was observed. CT, caloric test; HSCC, vHIT results in horizontal direction semicircular canal; PSCC, vHIT result in posterior semicircular canal; ASCC, vHIT result in anterior semicircular canal.

In the vestibular function examination results in the PO group, the abnormality rate of cVEMP (18/27, 66.67%) and PSC (18/27, 66.67%) was the highest, followed by the oVEMP (15/27, 55.56%), caloric test (15/27, 55.56%), horizontal semicircular canal (8/27, 29.63%), and anterior semicircular canal (1/27, 3.70%). The differences in the vestibular function tests between the two groups are shown in Figure 1.

**Figure 2:**
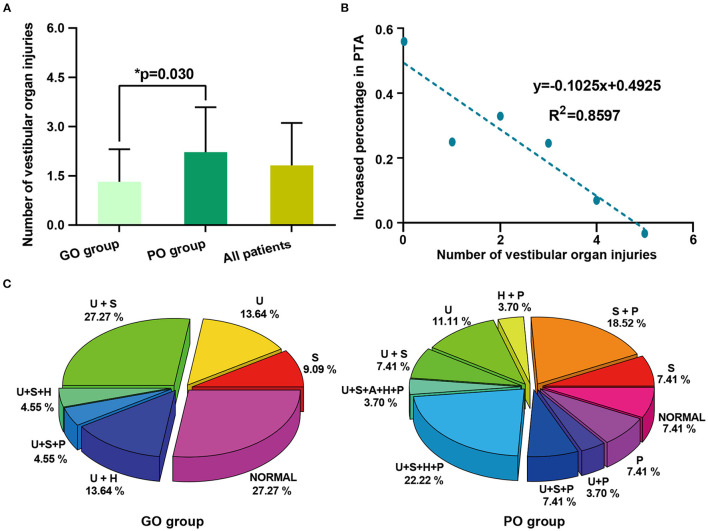
The comparison of vestibular organ injuries between the GO group and PO group. **(A)** The number of vestibular organ injuries between the GO group and PO group. **(B)** The linear fitting curve between the number of vestibular organ injuries and the average increased percentage in PTA. Each point on the figure was not a single patient, but the average increased percentage in PTA of all patients in the vestibular organ injury group. The results showed that the average increased percentage in PTA is linearly negatively correlated with the number of vestibular organ injuries (*R*^2^ = 0.8597, the linear equation was *y* = −0.1025x + 0.4925). **(C)** The specific modes of vestibular organs injuries between GO group and PO group. U, utricle dysfunction; S, saccule dysfunction; A, anterior semicircular canal dysfunction; H, horizontal semicircular canal dysfunction; P, posterior semicircular canal dysfunction. In the above statistical data on vestibular organ injuries, the results of the caloric test were not included.

The number of vestibular organ injuries was 1.82 ± 1.29 in all patients, with higher mean numbers in the PO group (2.22 ± 1.37) than in the GO group (1.32 ± 0.99). The difference in the number of vestibular organ injuries between the two groups is shown in Figure 2A. The linear fitting curve between the number of vestibular organ injuries and the average percentage increase in PTA is shown in Figure 2B. The results showed that the average increased percentage of PTA was linearly and negatively correlated with the number of vestibular organ injuries. (*R*^2^ = 0.8597; the linear equation was *y* = −0.1025*x* + 0.4925). The specific modes of vestibular organs injuries between GO group and PO group were shown in Figure 2C.

#### 3.2.3. Univariable and multivariable logistic regression analysis of prognostic factors for hearing recovery

Univariable logistic regression analysis of clinical characteristics and vestibular function test revealed that there were significant differences in the initial hearing threshold (*P* = 0.016) and abnormal vHIT result in PSC (*P* = 0.001) between the GO and PO groups; there was no significant difference in gender, age, side of the affected ear, vestibular symptoms, delayed treatment, instantaneous gain value of the horizontal semicircular canal, regression gain value of the vertical semicircular canal, abnormal rates of oVEMP, cVEMP, caloric test, and vHIT in the anterior and horizontal semicircular canals between these two groups. Multivariable logistic regression analysis of the parameters with significant differences showed that the significance of initial hearing loss disappeared, and only PSC injury was an independent risk factor for prognosis [*P* = 0.002, Exp (B) = 33.009]. These are listed in Table 2.

### 3.3. Abnormal vs. normal posterior semicircular canal function

The sensitivity, specificity, and positive and negative likelihood ratios were calculated. The sensitivity of abnormal PSC function in predicting poor prognosis in patients with severe and profound ISSNHL was 66.67%, specificity was 95.45%, and positive and negative likelihood ratios were 14.65 and 0.35, respectively. Patients with abnormal PSC function had worse initial hearing loss and prognosis than those with normal PSC function (Table 3).

**Table 3:** Abnormal vs. normal posterior semicircular canal function.

	**Hearing outcome**		**Initial hearing loss**	**Hearing improvement**

	**PO group (*****n*** = **27)**	**GO group (*****n*** = **22)**		
Abnormal posterior canal	18	1	101.58 ± 15.62 dB	10.86 ± 15.75 dB
Normal posterior canal	9	21	86.08 ± 17.08 dB	34.50 ± 23.49 dB
	Sensitivity = 66.67%	Specificity = 95.45%	*P* = 0.002	*P* < 0.001
	NLR = 0.35	PLR = 14.65		

NLR, negative likelihood ratios; PLR, positive likelihood ratios.

## 4. Discussion

The incidence of ISSNHL is ~5–20/100,000 people per year, and it has been gradually increasing with recent reports that younger populations are being affected (1). The percentage of patients with severe and profound hearing loss is ~41–74.2% (16). Unilateral severe to profound hearing loss inevitably affects a person's spatial hearing and speech recognition abilities, particularly in a long-term noise environment. The function of the auditory center of the cerebral cortex may degenerate, which can have a serious impact on the life, work, and psychology of the patient (17). In general, most patients with severe and profound ISSNHL have a negative prognosis due to the severity of their hearing loss. Age, initial hearing level, vestibular symptoms, and treatment onset have been previously reported in the literature as relevant indicators of prognosis in patients with ISSNHL (8). However, in our study, we found no significant relationship between gender, age, affected side, vestibular symptoms, delayed treatment, and poor hearing outcomes in patients with severe and profound ISSNHL.

The presence of vestibular symptoms in patients with sudden deafness is often considered an influential factor in poor hearing recovery; however, Wen et al. (18) found that patients with profound ISSNHL have worse hearing improvement, regardless of the presence of vestibular symptoms. Yu and Li (19) conducted a large sample size study on vestibular symptoms and hearing outcomes in patients with sudden deafness using a meta-analysis and found that vestibular symptoms may be negatively associated with hearing recovery, except in the group treated with intra- tympanic corticosteroid injections. Each patient in the current study was treated with intra-tympanic dexamethasone injections, which may be one of the reasons for our inconsistency with the results of previous studies. Meanwhile, our study showed no significant effect of the time of delayed treatment on hearing recovery, which may be because most of our patients (46/49) underwent timely treatment within 2 weeks, which is the therapeutic response period of treatment (1).

Due to the close anatomical and developmental relationship between the cochlea and vestibule, patients with severe and profound ISSNHL often have abnormal vestibular function, in addition to more damaged cochlear hair cells that are more difficult to recover (20). In this study, 26 of the 49 patients (26/49, 53.06%) presented with vestibular symptoms, and 46 patients (46/49, 93.88%) presented with abnormalities in vestibular function. Almost all patients had abnormalities in the objective tests of vestibular function. Meanwhile, our results showed that the average number of vestibular organ injuries was higher in the PO group than in the GO group. This suggests that the greater the extent of inner ear damage in patients with ISSNHL, the worse the prognosis, which is consistent with the results of previous research (21).

Previous studies have shown that abnormal VEMP is indicative of poor prognosis regardless of the onset of vestibular symptoms in patients with sudden deafness (22, 23). Shih et al. found that abnormal caloric test results were significantly associated with poor prognosis in patients with sudden deafness and that CP values were significantly associated with hearing recovery in patients with abnormal caloric test (24). However, no correlation was observed in the current study between vestibular test abnormalities and the prognosis of patients with severe and profound ISSNHL. Liang et al. (25) investigated the relationship between vestibular function and prognosis in patients with sudden deafness using a battery of vestibular function tests and showed that VEMPs may be a valid predictor of prognosis. While the results of the caloric test and vHIT test had no significant effect on hearing recovery, they did not correlate the abnormal results of the three semicircular canals on the affected side in vHIT with prognosis separately. However, the results of Guan et al. (26), in contrast to those of Liang et al. (25), revealed that the prognosis of patients with ISSNHL was only related to horizontal semicircular canal function impairment, but the study did not group the patients according to the degree of initial hearing loss.

Recently, Seo et al. found that higher initial hearing impairment and PSC abnormalities were associated with poor hearing prognosis in patients with profound sudden deafness (27). Our study is partially consistent with these results; however, the present study targeted a subgroup of patients with severe and profound hearing deafness, and the results of the prediction model showed that only abnormal PSC was an independent risk factor for poor prognosis in this subgroup. We found that patients with PSC injury had higher initial hearing impairment; therefore, the degree of initial hearing loss before treatment may have a collinearity relationship with PSC dysfunction. It is indirectly associated with prognostic outcome through its association with PSC functional loss; therefore, the significance of initial hearing loss disappeared in the multivariate analysis. PSC injury is likely to be a key factor in predicting prognosis. Almost all patients with ISSNHL with PSC impairment had a poor curative effect following treatment, with a specificity of 95.45% and a positive likelihood ratio of 14.65 for predicting poor outcome. Therefore, the results suggest that the vHIT can provide a preliminary assessment of patient prognosis and better respond to their consultation.

Currently, the pathogenesis of sudden deafness has not yet been ascertained, and microcirculatory disorders in the inner ear have been considered one of the main etiologies of ISSNHL (1). In previous reports, selective PSC dysfunction on vHIT was often associated with vestibulo-cochlear disorders such as vestibular neuritis and Meniere's disease, but it has rarely been mentioned in ISSNHL (28, 29). In 2005, Rambold et al. (30) found that 53% of patients with ISSNHL with vestibular lesions had a characteristic vestibulocochlear lesion pattern with combined injury of the cochlear and ipsilateral PSC, which may point to the vascular etiology of ISSNHL. In addition, Lee et al. and Yao et al. investigated vestibular function impairment in patients with ISSNHL and found that the abnormality rate of PSC was significantly higher than that of the anterior semicircular canal and horizontal semicircular canal (31, 32). Recently, several studies hypothesized that the mechanism of abnormal PSC function associated with poor hearing recovery may be related to the fact that the cochlea and PSC share a common branch artery for blood supply (27, 33, 34).

The internal auditory artery is the terminal artery supplying the labyrinth of the inner ear and is divided into the anterior vestibular artery and the common cochlear artery. The latter is further divided into the main cochlear and vestibulo-cochlear arteries, which provide blood supply to the cochlea. The vestibulocochlear artery supplies the basal turn of the cochlea, utricle, saccule, and PSC simultaneously. When the microcirculation of the vestibular cochlear artery or common cochlear artery is impaired and the cochlear blood supply is reduced, PSC also faces the risk of ischemia, while the utricle and saccule still have some blood from the anterior vestibular artery to compensate (35–39). Studies in animal models have found that vestibular and cochlear hair cell ischemia from various causes for more than 30 min is likely to cause permanent damage (40, 41). Based on these studies, we hypothesized that patients with severe and profound ISSNHL with abnormal PSC function may have impaired vascular supply to the vestibule and cochlea and that ischemia of the branches of the internal auditory artery supplying the cochlea and PSC may provide a possible explanation for the poor hearing prognosis in these patients.

Recently, Castellucci et al. (42) reported a case of a patient with multiple cardiovascular risk factors who had oriented the etiological hypothesis toward a possible common cochlear artery ischemia based on clinical symptoms and vestibular examination but found that only the PSC in vestibular end organs had imaging manifestations of post-ischemic fibrosis on steady-state gradient-echo MRI. Comacchio et al. (43) also reported a case of a patient with acute vestibular loss whose clinical manifestations and examinations mimicked inferior vestibular neuritis. The patient developed PSC ossification during follow-up. The authors speculated that the underlying etiology in this patient may have been posterior vestibular artery occlusion, although no other imaging manifestations of vestibular end-organ ischemia were detected on brain CT during the follow-up. In occlusion of the common cochlear artery or its inferior branches, the PSC appears to be at a greater risk of ischemia than other vestibular organs because of the lack of a dual blood supply. The imaging evidence in the above case report may strengthen the assumption of vascular pathomechanisms underlying the poor prognosis of patients with ISSNHL with PSC dysfunction.

However, despite the fact that the abnormal rate of PSC was the most prominent in the PO group, we found that the rates of utricle and saccule injury were also noticeable. This could be because patients in the PO group without PSC dysfunction seemed to have different patterns of vestibular damage, which may have increased the abnormal rates of oVEMP and cVEMP. If the above hypothesis is reasonable, early administration of blood-circulation-improving drugs or fibrinolytic drugs may improve the prognosis of patients with severe and profound sudden deafness with PSC injury; however, the effectiveness and efficacy of these drugs need to be further investigated.

However, there is one potential limitation to our study. When evaluating the prognosis of patients with ISSNHL, we did not assess the speech audiometry of patients after treatment, and only used PTA as the sole standard. As emphasized in the article, the significant decline in hearing function in patients with severe and profound ISSNHL will bring many obstacles to social life, and the speech discrimination score is also critical in evaluating the quality of hearing function in life.

In conclusion, abnormal PSC function is an independent risk factor for poor prognosis in patients with severe and profound ISSNHL. Patients with severe and profound ISSNHL and PSC abnormalities have higher initial hearing impairment and poorer prognosis. Ischemia in the branches of the internal auditory artery supplying the cochlea and PSC may be the underlying mechanism of poor hearing prognosis in patients with ISSNHL with PSC abnormalities.

## Data availability statement

The raw data supporting the conclusions of this article will be made available by the authors, without undue reservation.

## Ethics statement

The studies involving human participants were reviewed and approved by the Institutional Ethics Committee of Xinhua Hospital, Shanghai Jiao Tong University (NM: XHEC-D-2022-259). The patients/participants provided their written informed consent to participate in this study.

## Author contributions

JC, MD, and JY contributed to the study design. XM, JS, and QinZ preformed VEMPs, caloric test, and vHIT. YY and DG contributed to statistical analysis and manuscript draft. All authors helped to perform the analysis and to revise the manuscript with constructive discussions. All authors contributed to the article and approved the submitted version.
